# ChemReco: automated recognition of hand-drawn carbon–hydrogen–oxygen structures using deep learning

**DOI:** 10.1038/s41598-024-67496-7

**Published:** 2024-07-25

**Authors:** Hengjie Ouyang, Wei Liu, Jiajun Tao, Yanghong Luo, Wanjia Zhang, Jiayu Zhou, Shuqi Geng, Chengpeng Zhang

**Affiliations:** School of Informatics, Hunan University of Chinese Medicine, Changsha, 410208 Hunan People’s Republic of China

**Keywords:** Deep learning, SMILES coding, Image recognition, Convolutional neural network, Hand-drawn chemical molecular structure, Chemical molecular structure recognition, Encoder-decoder, Chemistry, Mathematics and computing

## Abstract

Chemical molecular structures are a direct and convenient means of expressing chemical knowledge, playing a vital role in academic communication. In chemistry, hand drawing is a common task for students and researchers. If we can convert hand-drawn chemical molecular structures into machine-readable formats, like SMILES encoding, computers can efficiently process and analyze these structures, significantly enhancing the efficiency of chemical research. Furthermore, with the progress of educational technology, automated grading is gaining popularity. When machines automatically recognize chemical molecular structures and assess the correctness of the drawings, it offers great convenience to teachers. We created ChemReco, a tool designed to identify chemical molecular structures involving three atoms: C, H, and O, providing convenience for chemical researchers. Currently, there are limited studies on hand-drawn chemical molecular structures. Therefore, the primary focus of this paper is constructing datasets. We propose a synthetic image method to rapidly generate images resembling hand-drawn chemical molecular structures, enhancing dataset acquisition efficiency. Regarding model selection, the hand-drawn chemical molecule structural recognition model developed in this article achieves a final recognition accuracy of 96.90%. This model employs the encoder-decoder architecture of EfficientNet + Transformer, demonstrating superior performance compared to other encoder-decoder combinations.

## Introduction

Chemical molecular structures play a crucial role in chemical education and academic communication. Compared to textual language, chemical molecular structures can represent chemical knowledge more directly and conveniently. When chemists and related researchers record and share molecular structures, hand-drawn diagrams are the most intuitive method. However, converting these diagrams into digital formats suitable for computation, retrieval, or storage typically requires manual input or specialized software for editing, a process that is both time-consuming and prone to errors. Moreover, with the development of educational informatization, automatic grading has become increasingly widespread. In chemistry exams, converting students' handwritten chemical molecular structures into machine-readable formats for automatic correctness assessment by machines would greatly benefit teachers.

Currently, the common methods for inputting chemical molecular structures into computers mainly rely on traditional click-and-drag interaction methods, which lack convenience and efficiency. With the large-scale development of touchscreen devices, handwriting-based input methods have significantly impacted human–computer interaction. Therefore, when we write chemical molecular structures on paper or a handwriting board, converting them into machine-readable data formats allows computers to process and analyze these structures. This form of input is much more convenient, thereby greatly improving the efficiency of chemical research. The process of automatically extracting molecules from images of chemical molecular structures and transforming them into machine-readable formats is known as optical chemical structure recognition. Many studies in this area depend on rule-based methods. These methods necessitate participants to possess significant professional knowledge and experience, integrating valence rules, chemical molecule bonds, patterns, and comprehensive visual features, along with domain knowledge, for effective identification. Despite the reliance on rule-based methods, certain intricate rules within chemical molecular structures pose challenges that these methods cannot adequately address for practical applications.

In recent years, the significant increase in computing power and the advent of the big data era have propelled the advancement of deep learning. Deep learning has demonstrated notable achievements in areas like computer vision and natural language processing. Within deep learning, the cornerstone is the datasets. Currently, there is a scarcity of datasets for hand-drawn chemical molecular structures, and the associated research is limited. Furthermore, the diverse writing styles of different individuals, poor image quality, and the absence of labeled data present challenges, rendering hand-drawn recognition a formidable task. This article addresses these challenges through the lens of deep learning, aiming to overcome the aforementioned difficulties and successfully identify and extract hand-drawn chemical molecular structures.

Many researchers worldwide have delved into image recognition in the chemistry domain, with a particular focus on chemical molecular structures. This area of study is known as Optical Chemical Structure Recognition (OCSR). However, there are noticeable gaps when it comes to recognizing hand-drawn chemical molecular structures. Hence, this study aims to bridge these gaps by integrating the OCSR method. It will specifically target the features of hand-drawn images and investigate automatic methods for recognizing and extracting hand-drawn chemical molecular structures.

The initial development of OCSR tools dates back to 1992. The first comprehensive tool, Kekule^1^, successfully identified chemical molecular structures from scanned images. In the same year, Ibison P and team introduced an OCSR software called Chemical Literature Data Extraction (CLiDE)^2^. Subsequently, an upgraded version, CLiDE Pro, was released^3^. Meanwhile, Optical Character Recognition (OCR) was making strides in various research areas. Researchers with expertise in mathematical OCR applied their knowledge to create an automated optical chemical structure recognition system, ChemInfty^4^. Its purpose was to identify chemical structure images in Japanese patent applications.

However, the mentioned studies relied on rule-based methods. In chemistry, the multitude of rules, coupled with the lack of comprehensive embedded rules, posed limitations. Rule-based systems tended to struggle with images featuring complex characteristics, blur, and noise^5^. Consequently, in response to the swift progress of machine learning technology, particularly deep learning algorithms, scholars both domestically and internationally have explored the application of deep learning algorithms for image recognition in the chemical domain.

Researchers in the field utilize segmentation models based on U-Net to extract chemical molecular structures. Subsequently, they employ attention mechanisms and Grid Long Short-Term Memory network (Grid LSTM) to identify these structures. The primary objective is to segment molecular structures from documents and predict chemical structures from the segmented images^6^.

In recent years, the DECIMER project for chemical image recognition, employing encoder-decoder architecture, has gained prominence^7^. This project also explores selected machine-readable encodings. Experimentation reveals that DeepSMILES^8^ encoding outperforms SMILES^9^ encoding, and SELFIES^10^ encoding surpasses DeepSMILES encoding in terms of recognition accuracy. A year later, researchers augmented the DECIMER project, resulting in DECIMER1.0^11^. This iteration improves the internal structure of the encoder-decoder, ultimately producing SELFIES encoding.

Simultaneously, the Img2Mol system was introduced^12^. The network learning proposed by the authors aims to accurately predict the CDDD^13^ embedding of the depicted chemical. They utilize a pre-trained CD decoder to obtain the SMILES encoding. Given the success of Transformers in various image-related fields, researchers have transformed images of organic chemical molecular structures into machine-readable forms using the Transformer architecture^14^. It has been demonstrated that the transformer-based network performs exceptionally well on simulation data provided by the authors, achieving high performance.

Researchers globally have engaged in extensive studies on image recognition in the chemistry field, with a focus on recognizing hand-drawn images. However, due to the intricacy of hand-drawn images, there has been relatively limited research. Chemistry encompasses four common text expression forms: chemical elements, chemical molecules, chemical molecular structures, and chemical equations. To recognize handwritten chemical symbols, some researchers have proposed a method based on Hidden Markov Model (HMM)^15^. Subsequently, they introduced a new two-stage classifier to enhance the initial method^16^.

Concerning handwritten chemical equations, as early as 1999, Ramel et al.^17^ proposed an offline method for identifying handwritten chemical equations in documents. This method extracts structured representations from hand-drawn images, generating results saved in vector form using specific knowledge. Yang et al.^18^ suggested an online handwritten chemical equation recognition method, employing a two-level algorithm for equation identification. Wang et al.^19^ later improved this method. Some researchers also utilized deep learning methods, proposing an end-to-end trainable system for recognizing handwritten chemical equations^20^. This system adopts the CNN + RNN + CTC framework.

In the realm of chemical molecular structural recognition, Ouyang^21^ developed a novel sketch-based system for interpreting hand-drawn organic chemical molecular structures. To assist chemical researchers, Sun et al.^22^ proposed a system for identifying chemical molecular structural sketches on smart mobile devices. The rise of deep learning has attracted researchers, with Zheng et al.^23^ utilizing VGGNet 19 to identify offline handwritten chemical organic ring structures.

Recently, Weir et al.^24^ created an offline hand-drawn hydrocarbon structure identification tool—ChemPix. This tool recognizes hand-drawn hydrocarbon structures and converts them into machine-readable SMILES codes. Additionally, the authors employed RDKit and various methods such as image augment, degradation, and background addition to generate large-scale synthetic datasets for auxiliary training. Compared to manual hand-drawing of chemical molecular structures, the synthetic image generation method significantly reduces the time-consuming manual effort.

This article primarily focuses on the automatic recognition of hand-drawn chemical molecular structures using deep learning. The initial step involves expanding and enhancing the datasets related to hand-drawn chemical molecular structures. Additionally, the paper adopts the widely used encoder-decoder architecture for image description tasks. It takes hand-drawn chemical molecular structural images as input and produces their corresponding SMILES code as output. The results obtained on the test set are notably positive, demonstrating higher accuracy compared to related studies.

## Materials and methods

### SMILES coding

The Simplified Molecular Input Line Entry System (SMILES) is a chemical symbol system specifically created for modern chemical information processing. It relies on the principles of molecular graph theory, employing a straightforward and natural syntax to describe chemical structures^9^. It is designed to be compatible with computer systems. However, due to factors like the rotation of chemical bonds and encoding starting from different atoms, one chemical molecular structure may correspond to multiple SMILES codes, posing challenges in subsequent identification processes. Therefore, this study concludes by converting the identified SMILES codes into standardized forms. Canonical SMILES encoding is generated following a strict set of rules and conventions to ensure the consistency and uniqueness of the encoding.

### RDKit

RDKit is an open-source chemical information toolkit widely applied in chemical reaction prediction, drug design, chemical molecular structure coding conversion, and related fields. It offers API interfaces in two languages, Python and C +  + , making it versatile across multiple operating systems. Leveraging the robust capabilities of the RDKit toolkit in the field of chemistry, this article utilizes RDKit for tasks such as molecular drawing and coding conversion of chemical molecular structures.

### Construction of hand-drawn chemical molecular structure data set

The task involves describing hand-drawn chemical molecular structures, which is essentially about converting images of these structures into machine-readable SMILES codes. To achieve this, we need extensive data for end-to-end deep learning models. Hence, a crucial part of this project is to enhance and broaden the datasets of hand-drawn chemical molecular structures. We collect SMILES encoding data from sources like PubChem, ZINC, GDB-11, and GDB-13 databases, focusing on C, H, and O atoms, and structures with up to 1 ring.

### Realistic hand-drawn chemical molecule structural data set

To ensure dependable datasets, this study employs RDKit's MaxMin algorithm for filtering SMILES encoding. The MaxMin algorithm helps select a subset from the entire molecular library, ensuring that the subset is highly diverse and effectively represents the chemical space of the original library. Subsequently, 670 SMILES codes were chosen and translated into chemical molecular structures.

**Figure 1:**
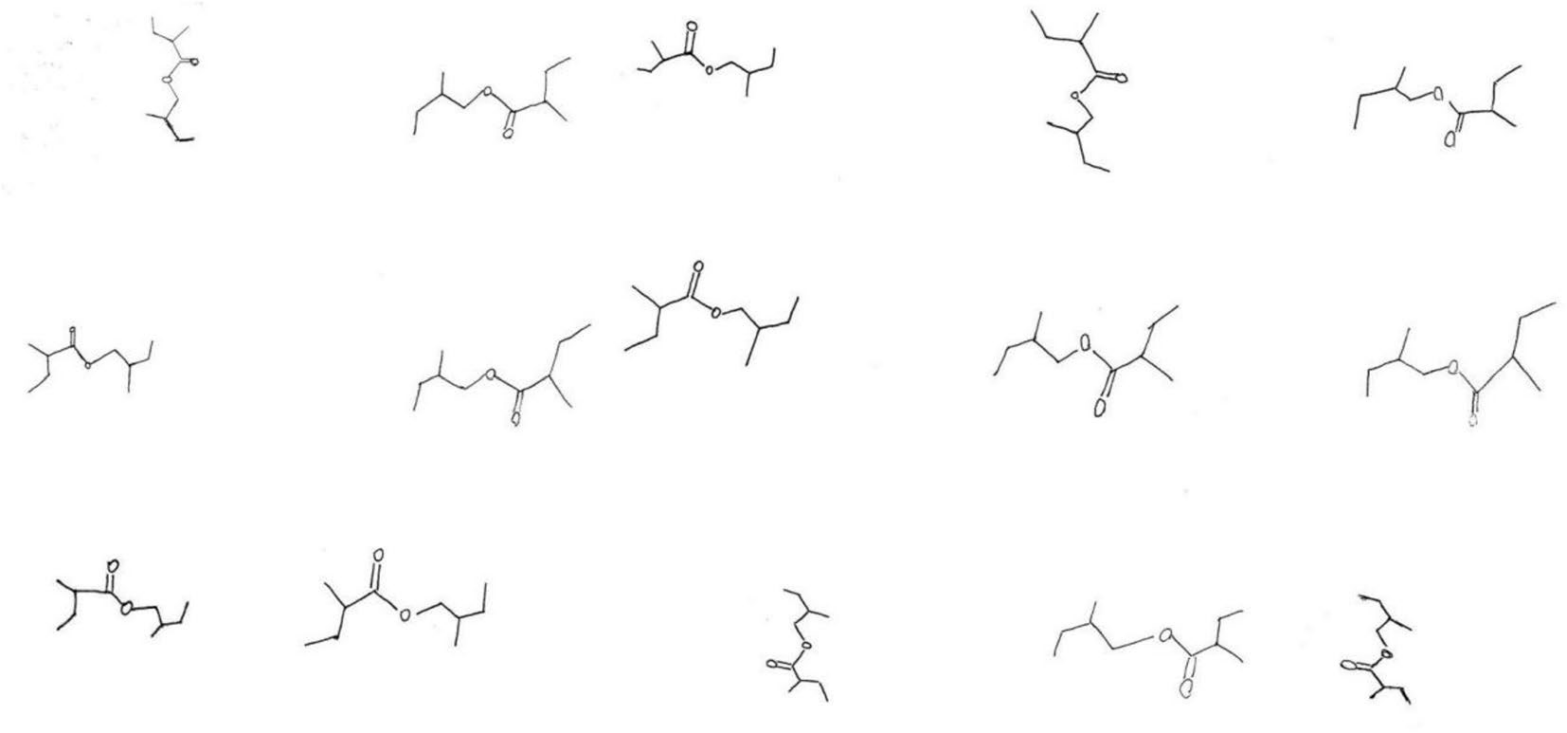
Here is a hand-drawn image of the structure of a chemical molecule. This chemical molecular structure is SMILES encoded as CCC(C)COC(= O)C(C)CC.

Each structure was drawn by multiple volunteers, resulting in a total of 2,598 pictures. The datasets dictionary includes symbols such as "#," "(", ")", "1," " = ", "C," "O," "c," "o," and the longest SMILES code has a length of 30. Figure 1 displays the hand-drawn chemical images in this article.

### Diffusion model-generated data set

Collecting real hand-drawn chemical molecular structures manually is time-consuming. Therefore, quick methods for obtaining datasets are necessary. With the growing use of AIGC in painting generation applications, researchers are increasingly applying it to various fields. In this study, the stable diffusion method^25^ is employed, based on the open-source stable-diffusion-v1-4 pre-training model, for image-to-image tasks. From the collected SMILES codes, a subset is selected and input into RDKit. Through image augmentation and degradation, a large number of standardized chemical molecular structure images are generated. By adjusting the image intensity and using prompts like "A pencil sketch of C6H6, 1024 × 1024 pixels, color, without charge distribution," etc., representative new hand-drawn chemical molecular structure images are generated. This approach preserves the original image features while introducing a certain level of uncertainty, resulting in new images with diverse styles, colors, or shapes.

### Synthetic chemical molecule structural data set

Training deep learning models with datasets from the two mentioned methods is insufficient and may lead to model overfitting due to the limited variety of real hand-drawn chemical molecular structures. Unfortunately, creating real hand-drawn structures is a challenging and time-intensive task. To address this, the study considers using synthetic images to expand the datasets.

**Figure 2:**
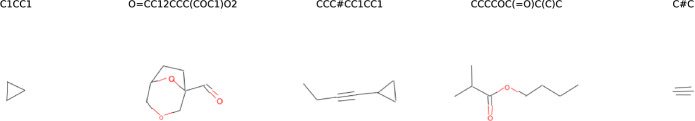
Modify the source code of the chemical molecule structural image drawn by RDKit.

Initially, modifications are made to the source code of RDKit to introduce random keys, character width, length, and angle. This results in the generation of a series of images resembling real hand-drawn chemical molecular structures, referred to as synthetic images, through SMILES coding. Figure 2 illustrates these synthetic images.

**Figure 3:**
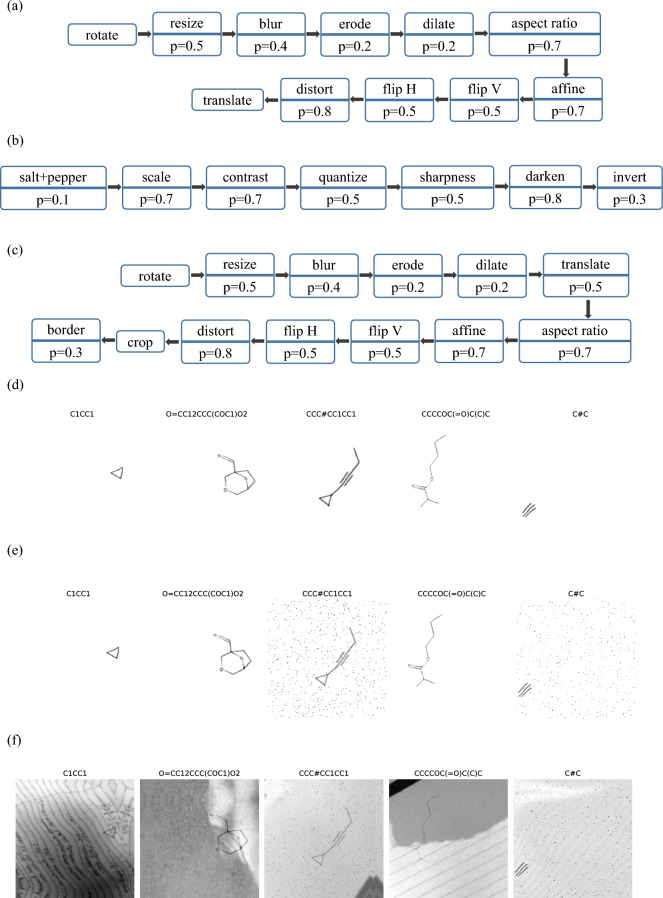
(**a**) Molecular image augments operations. (**b**) Molecular image degradation operations. (**c**) Background image augment operations. (**d**) The augmented chemical molecular structure image of Fig. 2 image. (**e**) Chemical molecular structure image after degradation of Fig. 3d image. (**f**) Chemical molecule structural image after adding background to Fig. 3e.

Subsequently, OpenCV is employed to conduct a sequence of image augment operations on the synthetic images to introduce noise and simulate real hand drawing. Random transformations are applied to the images. Figure 3a illustrates the sequence of image augment operations, and the augmented example image is presented in Fig. 3d (where "p" denotes operating with probability p).

Moreover, the paper carries out a degradation operation on the augmented images to simulate the loss and deformation observed in real hand-drawn images under various scenarios. Figure 3b illustrates the sequence of image degradation operations, and the degraded example image is displayed in Fig. 3e.

Finally, acknowledging that real hand-drawn chemical molecular structures may have diverse backgrounds, the study introduces random backgrounds to the images after the augment and degradation steps. The background image undergoes augment before being added, as depicted in Fig. 3c. Notably, if the background is not enhanced, the model might become overly accustomed to the background structure used during training, potentially learning to eliminate it from images. This can lead to poor generalization when exposed to images with backgrounds different from those encountered during training. Figure 3f showcases the image after incorporating the background.

Converting a SMILES code into the synthetic images described above takes approximately one second. In contrast, manually drawing a chemical structure and then processing it takes 2–3 min. Therefore, using this synthetic image method significantly reduces the time cost.

Based on the above operations, a large number of synthetic images can be quickly generated, significantly increasing the dataset size. For clarity, the various synthetic image generation methods are described as follows:

1. Firstly, by modifying the RDKit source code, random variations in bonds, character width, length, and angle are introduced (referred to as RDKit).
2. Based on the first method, OpenCV is used for image augmentation as shown in Fig. 3a (referred to as RDKit-aug).
3. Building on the second method, image degradation is performed on the augmented images as shown in Fig. 3b (referred to as RDKit-aug-deg).
4. Based on the second method, backgrounds processed as shown in Fig. 3c are randomly added to the images (referred to as RDKit-aug-bkg).
5. Building on the third method, backgrounds processed as shown in Fig. 3c are randomly added to the images (referred to as RDKit-aug-bkg-deg).

In summary, the dataset used in this study consists of two parts: real hand-drawn images and synthetic images. A large number of SMILES codes are obtained from the PubChem, ZINC, GDB-11, and GDB-13 databases. These SMILES codes form a dataset of manually drawn chemical molecular structure images and synthetic images. In practice, synthetic images generated using the methods described in the "Synthetic chemical molecule structural data set" are further enhanced by a diffusion model to improve their similarity to real hand-drawn images. Finally, the image resolution is adjusted to PNG format for model training.

## Recognition network of hand-drawn chemical molecular structural

### Image feature extraction

Convolutional Neural Networks (CNNs) are often used to extract features from images. In the second-to-last layer of the network, the features are kept for later use. To improve model accuracy, this paper evaluates ResNet and EfficientNet.

ResNet is a deep neural network proposed by He et al. in 2015. It aims to solve the problems of vanishing and exploding gradients during deep network training. ResNet has a hierarchical structure with residual connections, stacking residual blocks. Each block includes several convolutional layers and Batch Normalization. ResNet performed very well on the ImageNet dataset, achieving higher classification accuracy than earlier deep neural networks. As a result, ResNet is widely used in many computer vision tasks^26^.

**Figure 4:**
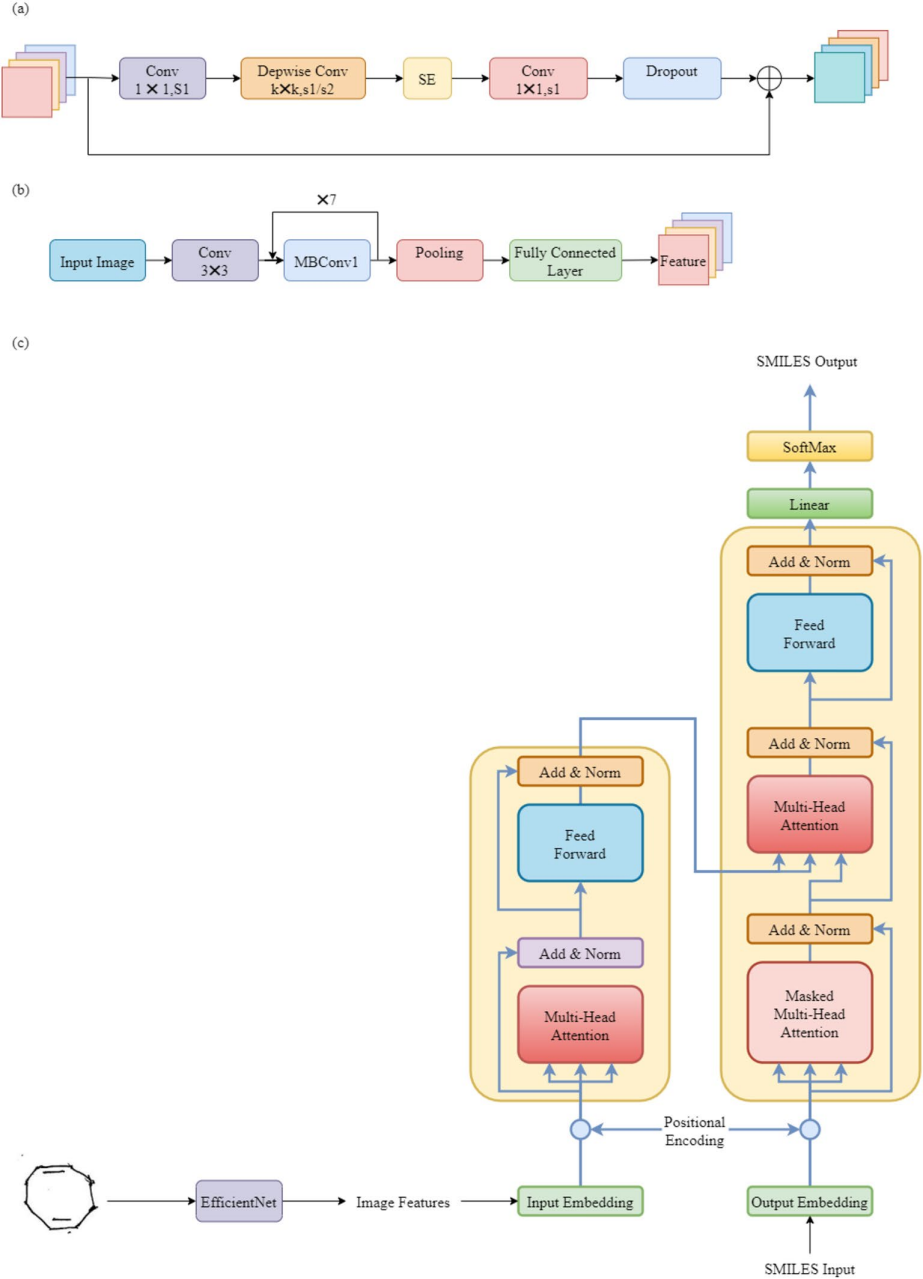
(**a**) The MBConv structure in EfficientNet; (**b**) The structure of the EfficientNet model; (**c**) The recognition network for hand-drawn chemical molecular structures.

EfficientNet, proposed by Google in 2019, is another efficient CNN architecture. It improves model performance by increasing network width, depth, and resolution. EfficientNet has multiple stages, each with several Mobile Inverted Bottleneck Convolution (MBConv) modules. The MBConv structure includes a 1 × 1 convolution (for dimension expansion with BN and Swish), a kxk Depthwise Conv (with Batch Normalization and Swish activation, where k is usually 3 × 3 or 5 × 5), an SE (Squeeze-and-Excitation) module, a 1 × 1 convolution (for dimension reduction with Batch Normalization), and a Dropout layer. The MBConv structure is shown in Fig. 4a. EfficientNet also performed well on the ImageNet dataset, exceeding models like ResNet-50 and ResNet-152 by over 3% with fewer parameters and computational needs^27^. The overall architecture is shown in Fig. 4b.

In this study, the model based on EfficientNet outperformed the one based on ResNet. Both EfficientNet and ResNet are designed for image classification, so the final layer features are used for classification. The last layer of the network is removed, and the extracted features are saved in a Numpy array for decoding. Additionally, the image feature extraction model uses transfer learning, importing pretrained weights from the ImageNet dataset and fine-tuning them on the hand-drawn chemical structure dataset.

### Decoding image features

After getting the image features from the encoder, a decoder is needed to output machine-readable SMILES codes. This study evaluates the performance of LSTM and Transformer as decoders.

LSTM (Long Short-Term Memory) is a type of recurrent neural network (RNN) used for sequence data. Compared to traditional RNNs, LSTM handles long sequences better and addresses vanishing and exploding gradient problems. Thus, LSTM performs well with long-sequence data^28^.

The Transformer is a neural network based on the self-attention mechanism, widely used in natural language processing (NLP). Proposed by Google researchers in 2017, it achieved top results in various NLP tasks. The Transformer does not use recurrent or convolutional structures. Instead, it relies on the self-attention mechanism to calculate dependencies between different positions in the input sequence. Each layer of the Transformer has a multi-head attention mechanism and a feedforward neural network. The multi-head attention calculates dependencies, while the feedforward network performs nonlinear transformations on the features.

In this study, the Transformer-based decoder outperformed the LSTM-based decoder, producing SMILES codes that better matched the chemical structures^29^.

The overall recognition network for hand-drawn chemical structures is shown in Fig. 4c.

### Training the models

The experimental setup in this article operates on the Ubuntu 18.04 operating system, featuring an Intel(R) Xeon(R) Gold 6130 CPU with 40 GB of memory. The GPU utilized is V100, boasting 32 GB of video memory. The deep learning framework employed is PyTorch 1.9.1. We used the Adam optimizer for training, with a default learning rate of 1e-4. The models were trained for 100 epochs with a batch size of 32. The evaluation metric was Exact Match, which directly checks if the SMILES codes of two chemical structures are exactly the same. As the name suggests, Exact Match has strict matching criteria compared to other metrics. The goal of this study was to ensure the model's recognition results perfectly match the ground truth labels. Therefore, Exact Match was the main criterion used to assess the models' performance.

## Results and discussion

### Synthetic image experiment

**Figure 5:**
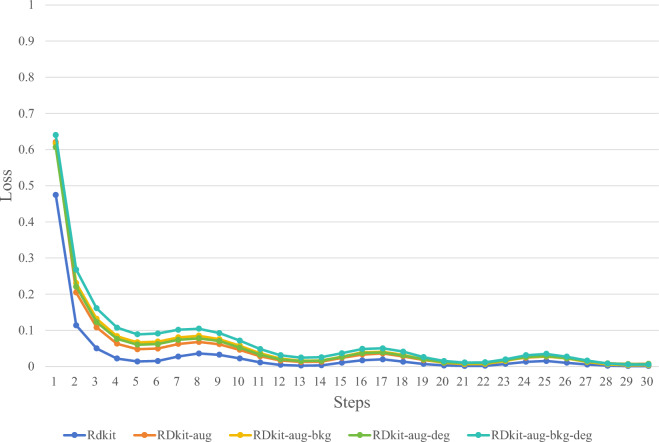
Comparison of Loss changes.

**Figure 6:**
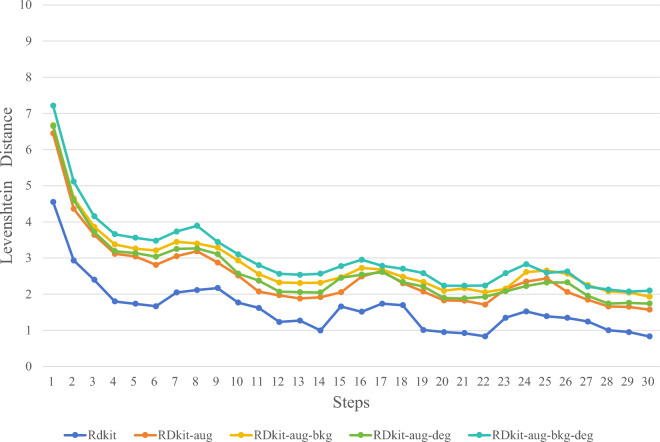
Comparison of Levenshtein distance changes.

**Figure 7:**
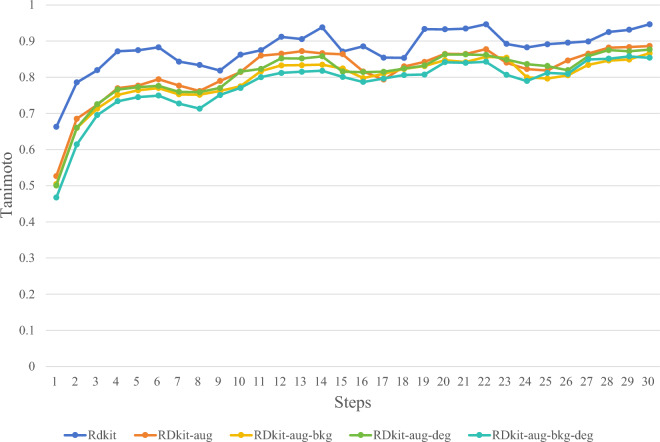
Comparison of Tanimoto coefficient changes.

**Figure 8:**
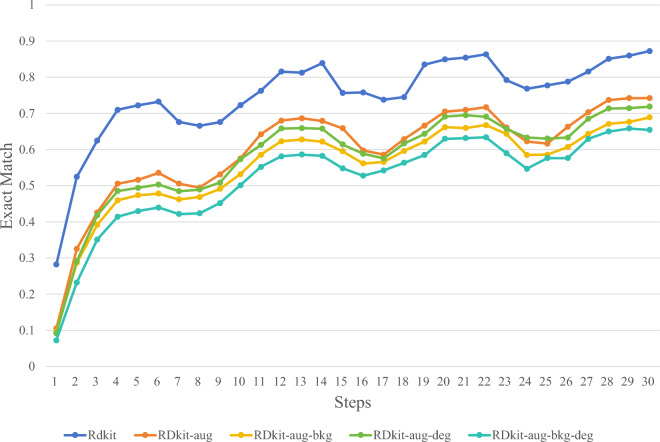
Comparison of exact match changes.

In this study, through ablation experiments, we experimented with various synthetic image methods mentioned in the "Synthetic chemical molecule structural data set" section to determine the optimal synthetic image generation method. Figures 5, 6, 7 and 8 illustrate several image synthesis methods for RDKit, RDKit-aug, RDKit-aug-bkg, RDKit-aug-deg, and RDKit-aug-deg-bkg, respectively. During the experiments, the change curve of loss value, Levenshtein Distance, Tanimoto coefficient, and Exact Match exact matching value on the verification set are observed.

**Figure 9:**
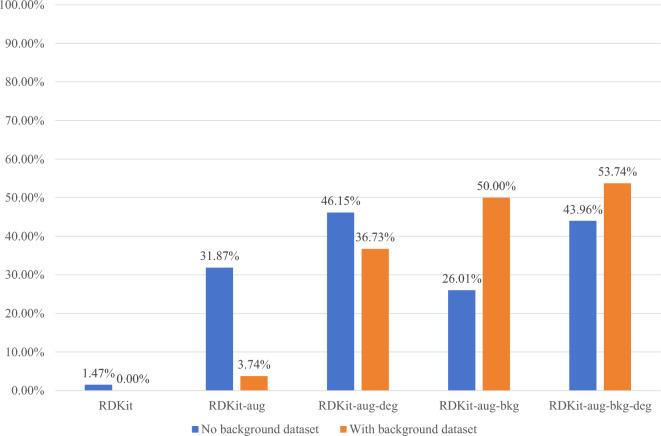
Accurate matching rate of different synthetic image methods on background and non-background data sets.

The experimental results above demonstrate that the model in this article can achieve commendable performance in the recognition of synthetic images among the various synthetic image methods. To provide a more precise validation of how the four steps of the synthetic image method in this article contribute to the identification of hand-drawn chemical molecular structures, Fig. 9 displays the experimental results of hand-drawn chemical molecular structures from the test set.

The utilization of image augments and image degradation operations results in improved recognition accuracy for hand-drawn chemical molecular structures, both in images with backgrounds and those without. This suggests that the model becomes more adept at discerning relevant information, even amid potential "interference" like background elements, focusing its attention on the chemical molecular structure.

Furthermore, the accuracy of images with backgrounds significantly increases after employing image augment and adding background compared to RDKit-aug. Providing molecular images with backgrounds for model learning allows it to acquire additional background-related knowledge. However, it is noteworthy that the accuracy of background-free images has decreased compared to RDKit-aug, indicating potential interference from the background images.

Among datasets using image augment, image degradation, and background addition, the background datasets achieve the best results. The accuracy on the background-free datasets is lower than that of RDKit-aug-deg.

In summary, it is observed that for the recognition of background-free datasets, RDKit-aug-deg attains the highest accuracy at approximately 46%. For the recognition of background datasets, RDKit-aug-bkg-deg achieves the highest accuracy at approximately 53%. Although the accuracy levels are not exceptionally high, it is noteworthy that during the model training process, the model did not acquire any knowledge about the structure of real hand-drawn chemical molecules. Solely relying on the knowledge from synthetic images yields an accuracy close to half.

This outcome underscores the inherent similarity between synthetic images and real hand-drawn chemical molecular structural images. Consequently, in subsequent experiments, this paper randomly employs the RDKit-aug-deg synthesis method and the RDKit-aug-bkg-deg synthesis method for RDKit images to potentially enhance the accuracy for both images with and without backgrounds.

### Synthetic image number experiment

To assess the model's performance across varying dataset sizes, this article conducted comparative experiments using different numbers of synthetic image datasets. These experiments aim to understand the model's sensitivity to data quantity, assess its generalization capabilities under different dataset sizes, and evaluate its ability to handle larger amounts of data.

**Figure 10:**
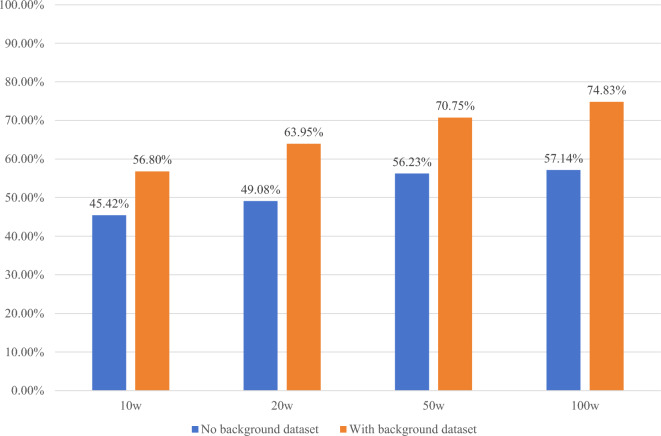
Accurate matching rate of different numbers of synthetic images on background and non-background data sets.

As illustrated in Fig. 10, the accurate matching rates are presented for 100,000, 200,000, 500,000, and 1 million synthetic images on the test set, both with and without background images. Table 1 displays the average exact matching rates on the test set.

**Table 1:** Average exact matching rate on the test set for different numbers of synthetic images.

Number of composite images	100,000	200,000	500,000	1,000,000
Exact match rate	49.40%	54.29%	61.31%	63.33%

It is evident that as the amount of data increases, the accurate matching rate shows improvement, both for images with and without background. When using 1 million synthetic images as the training and verification sets, the highest accuracy is achieved for both the datasets without background and the datasets with background.

**Figure 11:**
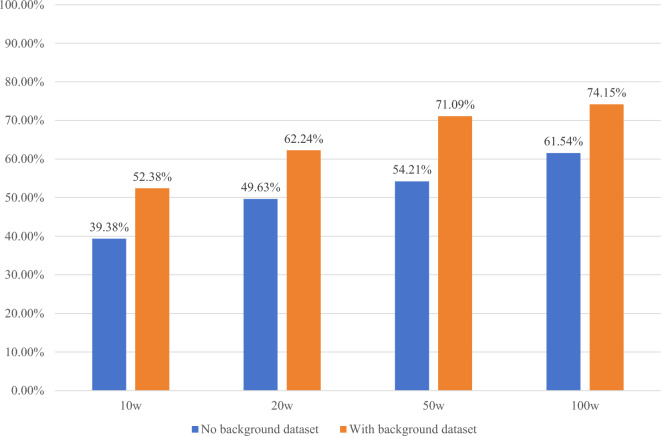
The verification set is real hand-drawn chemical molecular structure images and the accurate matching rate of different numbers of synthetic images on the background and non-background data sets.

Additionally, if the verification set is substituted with real hand-drawn chemical molecular structure images for testing, the obtained results are presented in Fig. 11 and Table 2.

**Table 2:** The verification set is real hand-drawn chemical molecular structure images and the average accurate matching rate of different numbers of synthetic images on the test set.

Number of composite images	100,000	200,000	500,000	1,000,000
Exact match rate	43.93%	54.05%	60.12%	65.95%

The experimental result indicates that substituting the verification set with real hand-drawn chemical molecular structure images does not yield significant differences from the previous results with synthetic images. This suggests that, at present, when the model is solely learning from synthetic images, merely replacing the verification set with real hand-drawn chemical molecular structures is insufficient.

To address this limitation, introducing some real hand-drawn chemical molecular structure images to the training set and allowing them to directly participate in the training process can enhance the model's capacity to learn from real-world examples. This approach has the potential to improve the accuracy of model recognition to a certain extent.

### Synthetic images hand-drawn image mixing ratio experiment

**Figure 12:**
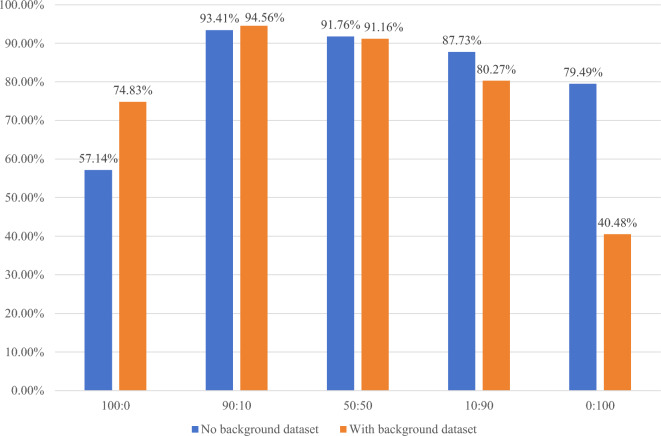
Different synthetic images: accurate matching rate of real hand-drawn image proportions on background and no-background datasets.

Hence, this article conducted comparative experiments with various proportions and identified the optimal balance between synthetic and real hand-drawn images. As depicted in Fig. 12, the synthetic chemical molecular structural images and real hand-drawn chemical molecular structures are used in training sets with ratios of 100:0, 90:10, 50:50, 10:90, and 0:100, respectively. The accurate matching rates of the resulting models on the background test set and the background-free test set are presented. Table 3 summarizes the average exact matching rates for the different ratios on the test set.

**Table 3:** Different synthetic images: average accurate matching rate of real hand-drawn image proportions on the test set.

Synthetic Image: Real hand-drawn image	100:0	90:10	50:50	10:90	0:100
Exact match rate	63.33%	93.81%	91.55%	85.12%	6 5.83%

The results indicate that when using all real hand-drawn chemical molecular structures in the training set (i.e., synthetic images: real hand-drawn chemical molecular structures at a ratio of 0:100), the accuracy rate is very low. This is because the datasets consist entirely of real chemical molecular structure images, and the model overfits these data, resulting in low accuracy on the test set.

Conversely, when the ratio is 100:0, indicating that the model's training set is entirely composed of synthetic images, and the model has not learned any knowledge from real hand-drawn chemical molecular structures, the accuracy is also not high.

At the ratio of 90:10, where 90% of the images are synthetic and 10% are real hand-drawn, the accuracy rate reaches 93.81%. Hence, the chosen composition is a 90:10 mix of synthetic images and real hand-drawn chemical molecular structure images.

### Encoder-decoder comparison experiment

**Figure 13:**
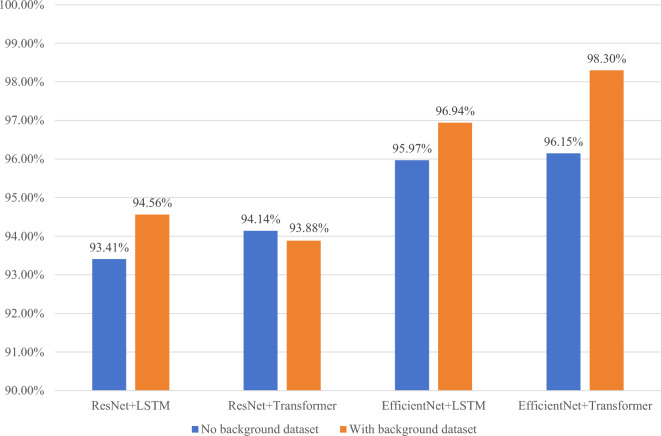
Accurate matching rate of different encoder-decoder combinations on background and non-background data sets.

In this experiment, the training set was curated from 1 million images, maintaining a ratio of 90:10 between synthetic chemical molecular structural images and real hand-drawn chemical molecular structural images, as it demonstrated the best performance in previous experiments. As illustrated in Fig. 13, it represents the exact matching rate on the test set with and without background when employing different encoder-decoder combinations. Table 4 provides the average exact matching rate on the test set for different encoder-decoder combinations.

**Table 4:** Average exact matching rate on the test set for different encoder-decoder combinations.

Encoder	Decoder	Average exact matching rate (%)
ResNet	LSTM	93.81
ResNet	Transformer	94.76
EfficientNet	LSTM	96.31
EfficientNet	Transformer	96.90

The results indicate that the model combination utilizing EfficientNet + Transformer achieves the most favorable recognition effect, boasting a final average accuracy of 96.90%.

Analysis of hand-drawn chemical molecule structural recognition results.

In the aforementioned experiment, it is evident that when opting for a ratio of 90:10 between synthetic images and real hand-drawn images, utilizing a total of 1 million images as the training set, and employing EfficientNet + Transformer as the encoder-decoder combination, this approach outperforms other combination methods. It achieves the best results in identifying hand-drawn chemical molecular structures, yielding an accurate matching rate of 96.90% on the test set.

### Comparison with related studies

There are currently limited studies on hand-drawn chemical molecular structure image recognition based on deep learning. In a related study^24^, a model combination of CNN + LSTM was utilized to convert hand-drawn hydrocarbon structure images into SMILES encoding. To enhance model accuracy, the author implemented a voting mechanism, achieving an accurate matching rate of 76% on the provided test set.

In comparison, the top-performing hand-drawn chemical molecule structural recognition model in this study was also evaluated on the test set provided in this article. It achieved a remarkable exact matching rate of 93%, significantly surpassing the accuracy reported in the mentioned paper. This outcome substantiates the advantages of the hand-drawn chemical molecule structural recognition model proposed in this article.

## Conclusion and future work

In addressing the challenge of small datasets for hand-drawn chemical molecular structures, this article undertook the collection and organization of datasets for such structures. Additionally, a synthetic image method was employed to generate hand-drawn chemical molecule structural images, offering advantages such as rapid generation and simplified labeling compared to manual drawings.

Furthermore, a deep learning model was constructed using an encoder-decoder architecture, enhancing recognition accuracy through data augmentation and transfer learning. The model structure was augmented with an attention mechanism, directing the model to focus on more pertinent locations. Ultimately, the article utilized the EfficientNet + Transformer model with a training set comprising synthetic images in a 90:10 ratio with real hand-drawn images. The model achieved an impressive accuracy rate of 96.90% on the test set consisting of real hand-drawn chemical molecular structural images. In comparison with related research, the proposed hand-drawn chemical molecular structure model in this article demonstrated significantly higher accuracy.

Given the rapid advancements in deep learning technology, its applications have expanded widely. Notable achievements have been made in areas like chemical reaction prediction and chemical property prediction. While research on chemical molecular structure is growing, there remains a gap in the study of hand-drawn chemical molecular structures. Given the evident importance of chemical molecular structures in the field of chemistry, future work could focus on adding more heteroatoms and complex ring structures, conducting experiments using the synthetic image method proposed in this article. Additionally, the identified SMILES codes can undergo further processing, such as creating a comprehensive database of chemical molecular structural s. This technology could find applications in education, automating the grading of chemistry test papers and determining the correctness of hand-drawn chemical molecular structures after converting them to SMILES encoding.

## Data Availability

The dataset used to support the findings of the study can be obtained from the corresponding author upon request. The code for this article is available at https://github.com/a-die/hdr-DeepLearning.
